# Quantitative Complexity Theory Used in the Prediction of Head-Up Tilt Testing Outcome

**DOI:** 10.1155/2021/8882498

**Published:** 2021-09-23

**Authors:** Paweł Krzesiński, Jacek Marczyk, Bartosz Wolszczak, Grzegorz Gielerak

**Affiliations:** ^1^Department of Cardiology and Internal Diseases, Military Institute of Medicine, Szaserów Street 128, Warsaw 04-141, Poland; ^2^Ontonix S.r.l., Via Campo Garibaldi 1, Como 22100, Italy

## Abstract

**Background:**

Head-up tilt testing (HUTT), a well-established tool in the diagnosis of vasovagal syncope, is time-consuming, and every provoked vasovagal reaction may result in consolidating the reflex mechanism. Therefore, identification of parameters that could shorten the duration of HUTT and prevent fainting is desirable. Quantitative complexity theory (QCT) may provide holistic information on the cardiovascular reaction in HUTT. The aim of the present article was to evaluate the prognostic value of complexity in comparison with traditional haemodynamic parameters (HR and BP) in predicting the HUTT outcome.

**Methods:**

Eighty-one healthy volunteers (74 men; mean age: 37.8 years) were included in this retrospective analysis of data collected within the project realized in Department of Cardiology and Internal Diseases, Military Institute of Medicine between January 2012 and October 2014. The subjects underwent HUTT, with beat-to-beat haemodynamic monitoring with a Niccomo™. The chosen haemodynamic parameters (including BP, HR, stroke volume, cardiac output, systemic vascular resistance) have been used in complexity analysis.

**Results:**

HUTT was positive in 54 (66.7%) study participants. The values of complexity were already higher in fainting subjects than those were in nonfainting ones 300 s before HUTT termination (HUTT_end), with a significant upward trend starting 150 s before (pre)syncope. An area under the curve (AUC) over 0.700 was observed for complexity from 120 s before HUTT_end, with a sensitivity of 63% and specificity of 78% at this time point. The prognostic value of complexity was superior to that of the HR and mean arterial pressure (MAP).

**Conclusions:**

Complexity has been shown to be a sensitive marker of cardiovascular haemodynamic response to orthostatic stress and proved to be superior over HR and BP in predicting HUTT outcomes.

## 1. Introduction

Syncope is a sudden loss of consciousness caused by transient cerebral global hypoperfusion with immediate and spontaneous recovery [1]. Vasovagal syncope (VVS) is a form of neurally mediated reflex syncope caused by a sudden decrease in blood pressure (BP) and/or heart rate (HR). The haemodynamic pattern of fainting determines the type of VVS; it can be vasodepressive, cardiodepressive, or mixed [2].

Head-up tilt testing (HUTT) is a well-established tool in the diagnosis of VVS. It allows to diagnose vasovagal syncope, usually benign in its clinical presentation [1]. However, it is time consuming, and such a provoked syncope may be an unpleasant event for a patient. Therefore, identification of parameters that could shorten the duration of HUTT and prevent final fainting is desirable.

The assessment of haemodynamic response in HUTT is usually based on *beat-to-beat* analysis of relatively easily accessible HR and BP. However, it allows only approximate explanation of complex physiological mechanisms involved in cardiovascular collapse while fainting. The approach to investigate HR and BP dynamics was developed in more advanced analyses, providing their mathematical derivatives, such as HR variability (HRV), BP variability (BPV), and baroreceptor sensitivity (BRS) [3–6].

Novel diagnostic tools, such as impedance cardiography (ICG) and plethysmography, enable continuous monitoring of other cardiovascular parameters, such as stroke volume (SV), cardiac output (CO), and systemic vascular resistance (SVR) [7–9]. They provide more detailed insight into haemodynamics than only HR and BP. However, there is still limited data regarding a combined analysis of set of haemodynamic parameters [7, 9–15]. From a practical point of view, a method merging haemodynamic data into one parameter would be the easiest to be applied in the evaluation of the onset of vasovagal reaction in clinical settings.

Quantitative complexity theory (QCT) stems from so-called complexity science [16] and has previously found some applications in medicine [17–20]. Complexity is a natural and physical property of every system and quantifies the amount of structured information contained therein. Conventional measures of complexity, such as Halstead complexity, cyclomatic complexity, time complexity, parametrised complexity, forecasting complexity, effective complexity, Kolmogorov complexity, a measure of algorithmic complexity, self-dissimilarity, U-rank and entropy, are not applicable when it comes to measuring the complexity of generic physical systems. A novel measure of complexity has been proposed by one of the coauthors (JM) [16, 21] as the amount of structured information contained in a system. It seems that it can provide quantitative and holistic information on the cardiovascular reaction in HUTT by merging multiple streams of haemodynamic data. The aim of the present article was to evaluate the prognostic value of complexity in comparison with traditional haemodynamic parameters (HR and BP) in predicting the HUTT outcome.

## 2. Methods

Eighty-one healthy volunteers (74 men and 7 women; mean age: 37.8 ± 4.7 years) were included in this retrospective analysis. The data were collected as a part of project no. 126/IWSZ/2007, funded by the Polish Ministry of National Defence and realized in Department of Cardiology and Internal Diseases, Military Institute of Medicine between January 2012 and October 2014. The project was approved by the appropriate ethics committee (no 11/WIM/2009) and performed in accordance with the ethical standards set out in the 1964 Declaration of Helsinki and its later amendments. The subjects aged 25–45 years, active soldiers, and without any chronic diseases were enrolled to this project. All participants provided written informed consent. Anonymity was ensured in all cases.

The subjects underwent HUTT, according to a modified version of the Italian Protocol [22] (passive phase of 15 min). After the stabilisation phase (5 min in the supine position), the subject was tilted to a position of 60–70 degrees. The passive phase of tilting was followed by the provocation phase of a further 15 min after the administration of 400 *μ*g of nitroglycerine sublingual spray. Test termination (supine restored) was made when the protocol was completed in the absence of symptoms, or there was the occurrence of syncope/presyncope. The examination was started before 2 PM in a fasting state in a quiet, warm, properly ventilated, and illuminated room.

### 2.1. Haemodynamic Assessment

Beat-to-beat haemodynamic cardiovascular response to tilting was evaluated by ICG, a modern, noninvasive method of haemodynamic monitoring. A Niccomo™ device (Medis, Ilmenau, Germany) integrated with a Tensoscreen™ module (Medis), dedicated to beat-to-beat BP assessment, was used. The final analysis included the following haemodynamic parameters: diastolic, systolic, and mean BP; pulse pressure; HR; pre-ejection period; left ventricular ejection time; stroke volume; cardiac output; Heather index; systemic vascular resistance; total artery compliance; and thoracic fluid content (described in detail in Table S1). All those parameters (besides mean BP as a derivate of diastolic and systolic BP) were used in the complexity analysis.

### 2.2. Quantitative Complexity Theory (QCT)

**Figure 1:**
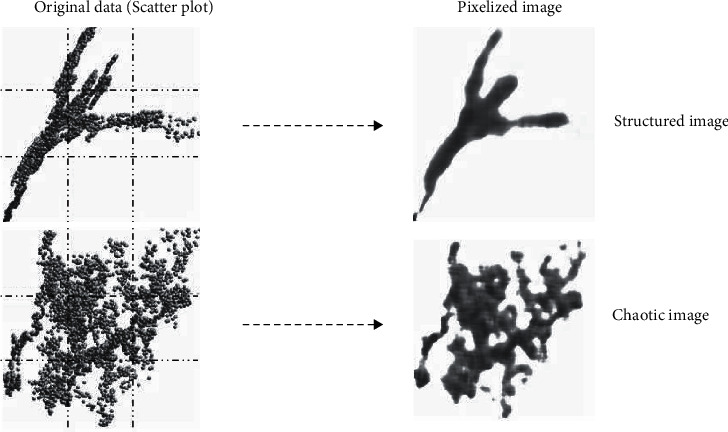
Examples of scatter plots and corresponding images. Images were obtained by subdividing the area of a scatter plot into pixels. The intensity of each pixel is proportional to the number of data points falling into it.

In our approach [16, 21], the complexity of a system with the state vector {*x*} of *N* components is defined as follows: *C* = *f*(*S*○*E*), where S represents an *N* × *N* adjacency matrix, *E* is an *N* × *N* entropy matrix, “○” is the Hadamard matrix product operator, and *f* is a spectral matrix norm operator. Given that *S* has no units, and because entropy is measured in bits, the units of *C* are also bits. This equation represents a formal definition of complexity, and it is not used in its computation. The adjacency matrix entries are 0 or 1, depending on the presence of interdependency between two state vector components. The presence and intensity of interdependency between the components of {*x*}, the so-called generalised correlation, is computed based on a proprietary algorithm that transforms scatter plots to images (Figure 1). Images are treated using entropy-based image processing techniques to determine if a given image is structured—that is, if two variables are correlated—or chaotic.

**Figure 2:**
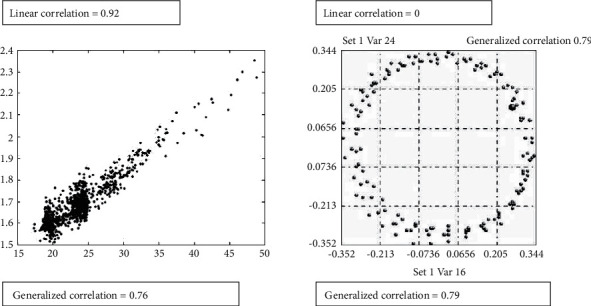
Examples of linear and generalised correlations.

This approach avoids the drawbacks of conventional linear techniques, which can, for example, miss significant correlations (see Figure 2). The main advantage of this approach is that it is independent of numerical conditioning of the data and the presence of outliers; moreover, it can identify the existence of correlation structures where conventional methods fail [21].

In the present analysis, a moving window of 100 samples of beat-to-beat haemodynamic cardiovascular parameters was applied. The data sampling frequency corresponded to HR frequency. This means that for an HR of 60 bpm, a window of 100 spans 100 s. The size of moving window was based on previous empiric observations (data not published).

### 2.3. Statistical Analysis

The obtained results were analysed statistically with Statistica 12.0 software (StatSoft Inc., Tulsa, OK, USA). The following time points were analysed: 300, 240, 210, 180, 150, 120, 90, 60, and 30 s before termination of the HUTT (HUTT_end). The distribution and normality of the data were assessed via visual inspection and the Kolmogorov–Smirnov test. Continuous variables were presented as means ± standard deviation (SD), and categorical variables were presented as absolute and relative frequencies (percentages). For comparative analysis (between subgroups with and without positive HUTT), the Student *t*-test or Mann–Whitney U-test was used, depending on the data distribution. To assess the predictive value of complexity, HR and mean arterial pressure (MAP) for the incidence of (pre)syncope during HUTT, the ROC curves were calculated for each of the time points listed above. A *p*-value of <0.05 was considered statistically significant.

## 3. Results

### 3.1. Basic Characteristics of the Study Group

The study participants were characterised by a mean BP of 117.9 ± 12.0/74.6 ± 7.7 mmHg and mean HR of 58.8 ± 8.9 bpm. They were free from chronic diseases and reported good physical fitness and regular physical training. The mean body mass index was 25.9 ± 2.6 kg/m^2^ (79 were nonobese). Only two subjects were current smokers. In 54 participants (66.7%), HUTT was positive and resulted in VVS (pre)syncope. No significant intergroup differences were noted (Table 1).

### 3.2. Comparison of Dynamic Changes of Complexity, Heart Rate, and Blood Pressure before HUTT Termination in Subgroups with and without Syncope

**Figure 3:**
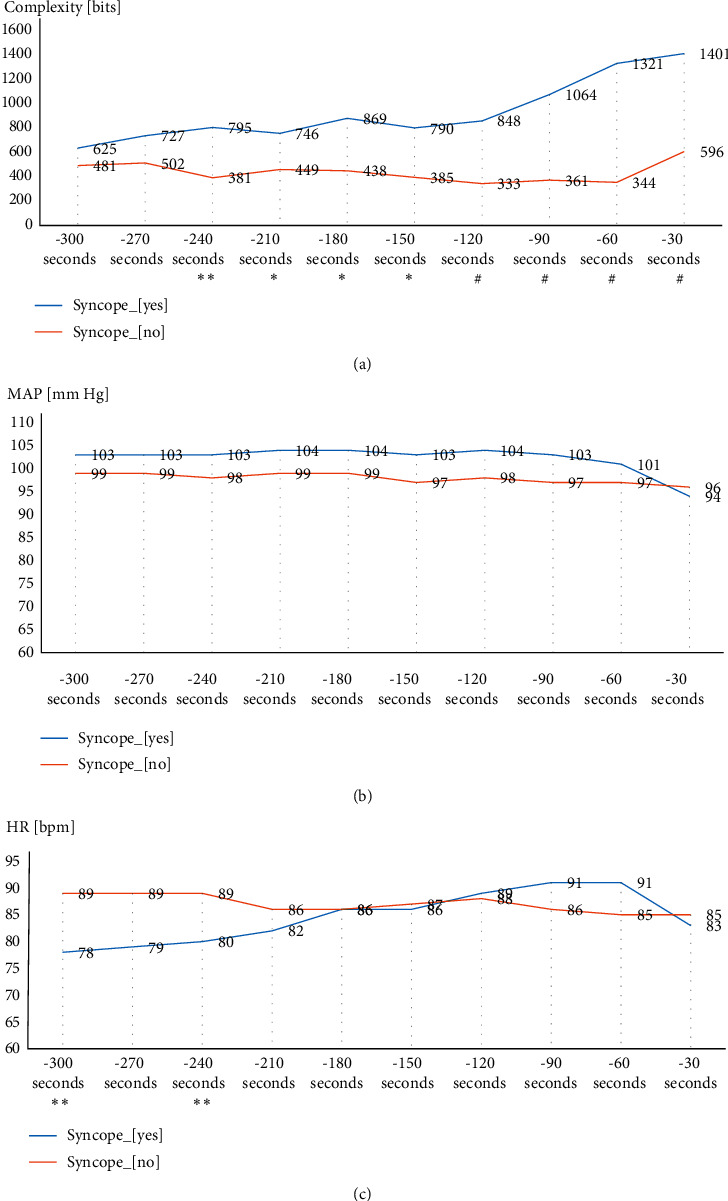
Intergroup comparison of complexity, mean arterial pressure (MAP), and heart rate (HR) values in the last 300 s before the termination of HUTT (^*∗*^*p* < 0.05, ^*∗∗*^*p* < 0.01, #*p* < 0.001).

The values of complexity were already higher in fainting subjects 300 s before HUTT_end, with a significant upward trend starting 150 s before (pre)syncope (Table 2; Figure 3). HR was higher in fainting subjects 240–300 s before HUTT_end, but then, the differences were not significant. No significant intergroup differences were observed for MAP.

### 3.3. The Prognostic Value of Complexity, Heart Rate, and Blood Pressure (ROC Analysis)

**Figure 4:**
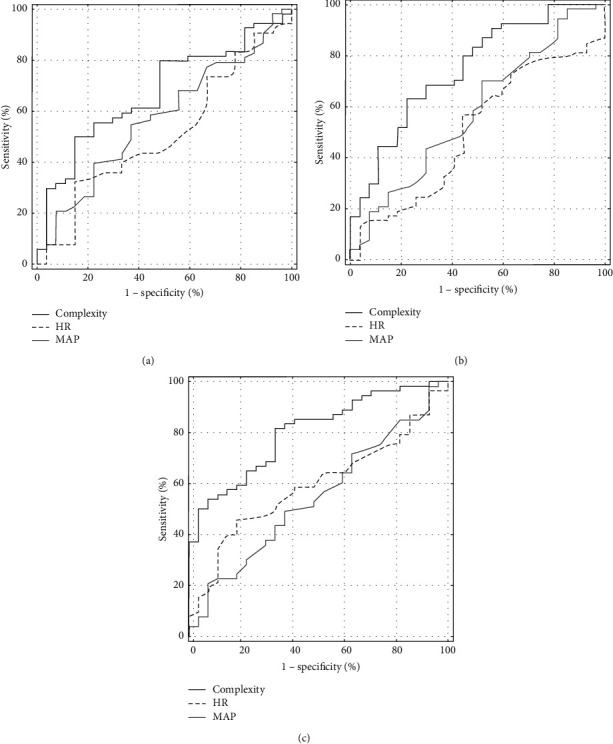
Comparison of ROC curves of complexity, mean arterial pressure (MAP) and heart rate (HR) for the chosen time points: 180 s (a), 120 s (b), and 60 s (c) before the termination of HUTT.

The results of the ROC analysis for complexity, MAP, and HR are presented in Table 3 and Figure 4. An area under the curve (AUC) over 0.700 was observed for complexity from 120 s before HUTT_end. Complexity performance in predicting (pre)syncope was expressed by sensitivity and specificity values of 63% and 78% at 120 s, 65% and 74% at 90 s, 82% and 67% at 60 s, and 69% and 74% at 30 s. Assuming a clinically acceptable sensitivity (>80%), the complexity revealed the following specificities at the indicated time points before HUTT_end: 52% at 120 s, 37% at 90 s, 67% at 60 s, and 60% at 30 s. On the other hand, for specificity over 80%, the following sensitivities were noted: 52% at 120 s, 50% at 90 s, 59% at 60 s, and 54% at 30 s (Table 4).

HR only presented a significantly higher AUC than complexity did at 300 s before HUTT_end, but the closer to the HUTT termination, the prognostic power of HR expired. There was also no intergroup difference for HR in time points from 210 s to 30 s before HUTT_end. The prognostic value of MAP was poor at all the analysed time points (max. 0.585).

## 4. Discussion

Our results suggest that complexity analysis based on QCT may be valuable in predicting the HUTT outcome, presenting additional value to HR and BP monitoring. Clinical application of QCT could avoid the necessity to impose a full syncopal event and make HUTT a less traumatic experience for the patient. In our study, HR and MAP were revealed to be inapplicable in predicting syncope, which can be explained by different haemodynamic patterns of vasovagal reaction that cannot be anticipated before HUTT (vasovagal, cardiodepressive, mixed).

Many subjects have little or no warning or prodromal symptoms before fainting [1]. Even if they occur, those symptoms preceding syncope for more than 3 min are nonspecific (headache, hot flashes, and palpitations) and related only to a slight reduction in BP [23]. A significant decrease of HR and/or BP is usually reported no longer than 1 min before fainting, when more specific symptoms may be present (nausea, asthenia, diaphoresis, vertigo, and blurred vision) [23]. The time point of 120 s provided clinically acceptable cutoffs for discontinuing the HUTT. If the identification of subjects with VVS is the priority, the cutoff 240.6 bits can be used (sensitivity >80% and specificity 52%), and if exclusion of other than vasovagal (i.e., malignant) nature of syncope is more clinical relevant, 523.7 bits should be applied (specificity >80% and sensitivity 52%).

Several earlier studies investigated haemodynamic predictors of syncope in the late phase of HUTT. Mereu et al. [24] derived the ratio between the RR interval and systolic BP (dRR/SBP) to predict syncope 44.1 ± 6.6 s in advance, with a sensitivity of 86.2% and specificity of 89.1% (AUC = 0.877). Virag et al. [10] evaluated the VVS prediction algorithm based on simultaneous analysis of RR intervals, SBP trends, and their variability. The best performance was a sensitivity of 97.6% and specificity of 88.2%, with a mean VVS prediction time of 2 min 26 s (median: 1 min 25 s). Bellard et al. [11] performed a detailed analysis of haemodynamics assessed by ICG to predict the outcome of the nitroglycerine–sensitised HUTT. The combination of cutoff values of several ICG parameters (i.e. preejection and rapid left ventricular ejection time, slow ejection time, peak amplitude of first derivate, cardiac index) during the last 5 min resulted in a sensitivity of 76% and specificity of 87%.

There were also some attempts to predict the HUTT result based on resting supine measurements and the early phase of HUTT. Schang et al. [25] reliably predicted a positive HUTT with a sensitivity of 88% and specificity of 64% using neural networks to analyse the pretilting supine rest impedance waveform and its first derivate. Parry et al. [9] analysed supine ICG haemodynamic measures (cardiac index, enddiastolic index, and left ventricular work index) and derived the algorithm to identify positive HUTT with high sensitivity (93%) but very low specificity (17%). Gielerak et al. [12] analysed posttilting haemodynamic changes in the prediction of the HUTT result and identified the mean vascular resistance difference of <−10 dyn*∗*s/cm^8^ as an independent risk factor of syncope (sensitivity: 65% and specificity: 61%). Koźluk et al. [7] observed that positive HUTT results were related to a higher reduction of stroke volume (−27.2 ml vs. −9.7 ml; *p*=0.03) and cardiac output (−1.78 l/min vs –0.34 l/min; *p*=0.032), 5 min after tilting. Maier et al. [26] showed that HRV analysis within the first 5 min after tilting provided significant information on HUTT outcomes when time-domain parameters (standard deviation of differences for successive RR intervals) reached a sensitivity of 75% and specificity of 80%. In contrast, Klemenc and Štrumbelj [27] failed to prove that early HRV and BRS are sufficient to predict syncope in the HUTT.

Predicting syncope appears to be a difficult task because the cardiovascular response to upright posture depends on complex mechanisms. Faes et al. [13], using a concept of information processing, noted information domain decomposition of HR variability and mean cerebral blood flow velocity (CBFV) variability several minutes preceding presyncope. These phenomena may reflect both cardiovascular and cerebrovascular impairments, including weakening of baroreflex modulation and cerebral autoregulation. Porta et al. [14] evaluated multivariate autoregressive (MAR) model, including analysis of HR, BP, and respiration while modulating autonomic function (beta-adrenergic blockade, central sympathetic blockade, graded head-up tilt) and concluded that complexity indexes provide complementary information. HR complexity was found to be more related to vagal activity and BP complexity to sympathetic activity. The authors suggested that such an method might be used for early detection of the impairment of the autonomic nervous system. In another study, Porta et al. [15] confirmed that their approach provide quantitative indexes helpful in elucidating the effect of age on cardiovascular control in humans.

The application of QCT, an advanced mathematical model integrating several parameters into one marker, allowed for the prediction of the HUTT outcome with clinically acceptable power and timing.

The previous reports described above basically used post hoc, sophisticated, analytical methods. Their advantage is that most of them provide abundant data and explain, at least to some extent, pathophysiological background of syncope. The importance of detailed investigating physiological mechanisms of cardiovascular interactions accurately illustrates the studies such as that reported by Javorka et al. [28]. The authors clearly presented how multivariate time series analysis of HR and BP enable identifing interactions between two signal changes in the context of networks including other coupled signals.

The relevant advantage of QCT is the emerging possibility of real-time analysis and delivering patient monitoring software for mobile devices, such as smartwatches or tablets. QCT merges multiple streams of haemodynamic data into one parameter and provide quantitative and holistic information on the cardiovascular reaction to orthostatic challenge. However, it is also a potential disadvantage. Such an approach might be perceived as a “black-box,” missing a detailed insight into the mechanism of vasovagal reaction. Although the contribution of haemodynamic parameters might be derived from complexity profiles (Figure S1), this approach should be investigated in further studies and confronted with better validated methods. One should be also aware that complexity may rise in case of any stimulation of cardiovascular system. Therefore, respecting the rules of performing HUTT in quit, warm, properly ventilated, and illuminated room is of special importance. Moreover, the complexity analysis requires high-frequency (optimal beat-by-beat) acquisition of the parameters, which may be challenging in some clinical conditions.

### 4.1. Limitations

This study had some limitations. First, the present research was a retrospective analysis of a single-centre study with all the accompanying limitations inherent to such a design. Second, HUTT was performed in healthy volunteers without a spontaneous syncope history. The high occurrence of HUTT-induced syncope was probably related to accumulation of factors predisposing to orthostatic intolerance and specific for this study group, especially young age and higher than normal exercise training load. Therefore, our results are not conclusive of the diagnostic value of HUTT in general population. Moreover, the contribution of our approach to the clinical practice should be derived from further research in target populations, such as patients with a history of syncopal events. A comparison of QTC to other diagnostic methods discussed above while designing further studies could be very valuable.

The findings of intergroup differences in HR at 300 and 240 s before HUTT, as though relatively high AUC at 300 s before HUTT for HR, should also be commented. The causal relationship of this observation with the result of HUTT seems to be unlikely. Firstly, the results are inconsistent with the expectation that as syncope approaches, the prognostic power of the predictor increases. Secondly, the intergroup comparison of trends (Figure 3) shows that while HR at 300–240 s before HUTT was lower in fainting subjects than in nonfainting ones, already from 210 s before HUTT it increased and even exceeded the value for the second group. This intergroup difference in HR at 300 and 240 s before HUTT was most likely related to the fact that all subjects with negative test were exposed to nitroglycerine, which induced tachycardia, whereas some of those fainting ones ended the test in its passive phase.

We are also aware that the description of QCT in this article is limited, but the technology is proprietary by one of the coauthors (JM) and certain details cannot be disclosed.

## 5. Conclusions

Complexity has been shown to be a sensitive marker of cardiovascular haemodynamic response to orthostatic stress and proved its superiority over HR and BP in predicting HUTT outcomes. The predictive performance (sensitivity >80% and specificity >50%) of complexity even 2 min before syncope seems to be clinically acceptable. Beat-to-beat complexity analysis may be used to terminate HUTT before triggering a symptomatic vasovagal reflex with a high probability of correct diagnosis. The present results encourage validating the complexity method in other clinical settings and in prospectively designed trials.

## Figures and Tables

**Table 1:** Intergroup comparison of baseline characteristics.

Variable	Syncope_[yes] *n* = 54	Syncope_[no] *n* = 27	*p*
Female/male	51 (94%)/3 (6%)	23 (85%)/4 (15%)	0.162
Age (years)	38.1 ± 4.6	37.1 ± 4.8	0.368
Office HR (bpm)	58 ± 9	60 ± 9	0.541
Office SBP (mm Hg)	117 ± 10	119 ± 16	0.573
Office DBP (mm Hg)	75 ± 7	74 ± 9	0.738
BMI (kg/m^2^)	26 ± 3	26 ± 3	0.470
Creatinine (mg/dL)	0.94 ± 0.9	0.91 ± 0.13	0.139
Hemoglobin (g/dL)	14.5 ± 0.9	14.8 ± 1.1	0.279

Data presented as mean ± standard deviation (SD) and *n* (%), DBP: diastolic blood pressure HR: heart rate, SBP: systolic blood pressure, BMI: body mass index.

**Table 2:** Intergroup comparison of complexity, mean arterial pressure (MAP), and heart rate (HR) in the last 300 s before the termination of HUTT.

Time to HUTT_end (seconds)	Variable	Syncope_[yes] *n* = 54	Syncope_[no] *n* = 27
300	Complexity (bits)	625.6 ± 848.2	481.3 ± 462.3
	HR (bpm)	77.6 ± 13.0^*∗∗*^	88.9 ± 16.2^*∗∗*^
	MAP (mm Hg)	103.2 ± 16.5	99.1 ± 16.8

270	Complexity (bits)	726.5 ± 1077.4	501.9 ± 509.5
	HR (bpm)	79.4 ± 12.8	88.8 ± 16.9
	MAP (mm Hg)	102.8 ± 18.2	99.0 ± 17.6

240	Complexity (bits)	**795.4** **±** **1011.6**^*∗∗*^	**380.7** **±** **355.4**^*∗∗*^
	HR (bpm)	79.5 ± 13.4^*∗∗*^	88.8 ± 13.8^*∗∗*^
	MAP (mm Hg)	102.6 ± 17.9	98.0 ± 17.4

210	Complexity (bits)	746.4 ± 1142.9	449.1 ± 386.7
	HR (bpm)	82.0 ± 14.7	86.2 ± 17.0
	MAP (mm Hg)	103.8 ± 19.1	98.9 ± 17.4

180	Complexity (bits)	**868.9** **±** **1221.8**^*∗*^	**438.0** **±** **475.1**^*∗*^
	HR (bpm)	86.4 ± 17.5	86.3 ± 14.7
	MAP (mm Hg)	103.8 ± 19.0	98.9 ± 17.1

150	Complexity (bits)	**790.1** **±** **860.9**^*∗*^	**385.0** **±** **435.4**^*∗*^
	HR (bpm)	85.9 ± 15.3	86.5 ± 13.6
	MAP (mm Hg)	103.2 ± 19.1	97.6 ± 18.2

120	Complexity (bits)	**847.7** **±** **1006.1#**	**333.4** **±** **1006.1#**
	HR (bpm)	88.9 ± 17.5	87.9 ± 14.7
	MAP (mm Hg)	104.1 ± 18.7	98.1 ± 18.4

90	Complexity (bits)	**1063.5** **±** **1287.7#**	**360.8** **±** **268.7#**
	HR (bpm)	90.8 ± 19.5	86.4 ± 15.3
	MAP (mm Hg)	103.1 ± 18.8	97.1 ± 17.5

60	Complexity (bits)	**1320.9** **±** **1400.9#**	**344.3** **±** **245.2#**
	HR (bpm)	91.1 ± 21.6	85.1 ± 14.2
	MAP (mm Hg)	101.2 ± 19.9	97.4 ± 17.3

30	Complexity (bits)	**1401.3** **±** **1519.4#**	**596.4** **±** **1041.2#**
	HR (bpm)	83.3 ± 21.6	85.1 ± 14.2
	MAP (mm Hg)	93.9 ± 20.7	96.4 ± 19.8

Data presented as mean ± standard deviation (SD). HR: heart rate, HUTT: head-up tilt test, MAP: mean blood pressure. Statistically significant intergroup difference (syncope_[yes] vs syncope_[no]) is marked as ^*∗*^*p* < 0.05; ^*∗∗*^*p* < 0.01; #*p* < 0.001.

**Table 3:** Prognostic value of complexity, mean arterial pressure (MAP), and heart rate (HR) in the last 300 seconds before the termination of HUTT.

Time to HUTT_end (seconds)	Variable	AUC (95% CI)^*∗*^
300	Complexity (bits)	0.527 (0.393–0.661)
	HR (bpm)	0.713 (0.591–0.834)^0.026^
	MAP (mm Hg)	0.585 (0.452–0.718)

270	Complexity (bits)	0.576 (0.440–0.712)
	HR (bpm)	0.632 (494–0.770)
	MAP (mm Hg)	0.564 (0.429–0.698)

240	Complexity (bits)	0.683 (0.558–0.808)
	HR (bpm)	0.713 (0.594–0.833)
	MAP (mm Hg)	0.574 (0.441–0.707)

210	Complexity (bits)	0.566 (0.431–0.700)
	HR (bpm)	0.583 (0.447–0.718)
	MAP (mm Hg)	0.577 (0.448–0.706)

180	Complexity (bits)	0.673 (0.553)-0.793)
	HR (bpm)	0.510 (0.377–0.644)
	MAP (mm Hg)	0.573 (0.442–0.704)

150	Complexity (bits)	0.660 (0.539–0.781)
	HR (bpm)	0.520 (0.386–0.655)
	MAP (mm Hg)	0.578 (0.445–0.711)^0.050^

120	Complexity (bits)	0.747 (0.634–0.860)
	HR (bpm)	0.504 (0.369–0.638)^0.034^
	MAP (mm Hg)	0.578 (0.444–0.712)^0.106^

90	Complexity (bits)	0.736 (0.625–0,847)
	HR (bpm)	0.535 (0.405–0.665)^0.007^
	MAP (mm Hg)	0.572 (0.437–0.707)

60	Complexity (bits)	0.802 (0.707–0.897)
	HR (bpm)	0.590 (0.465–0.716)^0.015^
	MAP (mm Hg)	0.546 (0.414–0.677)^0.003^

30	Complexity (bits)	0.772 (0.660–0.883)
	HR (bpm)	0.525 (0.398–0.652)^0.014^
	MAP (mm Hg)	0.550 (0.417–0.683)^0.030^

^*∗*^Statistically significant intervariable difference (complexity vs HR/MAP); *p* value was presented in superscript. AUC: area under curve, HR: heart rate, HUTT: head-up tilt test, MAP: mean arterial pressure.

**Table 4:** The optimal cutoffs of complexity (column A) within last 300 seconds before the termination of HUTT, corresponding sensitivity and specificity (column B), specificity in term sensitivity over 80% (column C), and sensitivity in term specificity over 80% (column D).

time to HUTT_end (seconds)		Column A -optimal cutoff	Column B -sensitivity/specificity for optimal cutoff	Column C -cutoff (specificity) when sensitivity over 80%	Column D -cutoff (sensitivity) when specificity over 80%
300	Complexity (bits)	199.3	83%/26%	193.8 (26%)	689.2 (20%)

270	Complexity (bits)	231.7	78%/44%	194.3 (26%)	715.7 (30%)

240	Complexity (bits)	246.9	76%/56%	208.8 (48%)	611.5 (51%)

210	Complexity (bits)	231.7	78%/44%	188.5 (26%)	713.7 (26%)

180	Complexity (bits)	598.0	50%/85%	230.4 (41%)	591.1 (50%)

150	Complexity (bits)	319.1	61%/70%	184.2 (33%)	470.1 (48%)

120	Complexity (bits)	411.5	63%/78%	240.6 (52%)	523.7 (52%)

90	Complexity (bits)	456.5	65%/74%	130.8 (37%)	586.1 (50%)

60	Complexity (bits)	385.6	82%/67%	385.6 (67%)	584.2 (59%)

30	Complexity (bits)	545.7	69%/74%	371.7 (59%)	753.0 (54%)

HUTT, head-up tilt test.

## Data Availability

The data used to support the findings of this study are available from the corresponding author upon request.

## Supplementary Materials

Supplementary Materials Supplementary Materials include the list of haemodynamic parameters measured by impedance cardiography (Table S1) and examples of complexity profiles showing the breakdown of total system complexity into its components in terms of the percentage of contribution of each component (Figure S1).
