# Supplementary material for: Quantitative Complexity Theory Used in the Prediction of Head-Up Tilt Testing Outcome
Source: Cardiol Res Pract. 2021 Sep 23;2021:8882498. doi: 10.1155/2021/8882498 (PMC8486546; doi:10.1155/2021/8882498)
Supplement: Supplementary Materials — include the list of haemodynamic parameters measured by impedance cardiography (Table S1) and examples of complexity profiles showing the breakdown of total system complexity into its components in terms of the percentage of contribution of each component (Figure S1). [file 8882498.f1.docx]

**SUPPLEMENTARY MATERIAL.**

*Table S1. List of hemodynamic parameters.*

| **Parameter** | **Acronym** | **Unit of measure** |
| --- | --- | --- |
| Diastolic Blood Pressure | DBP | mmHg |
| Systolic Blood Pressure | SBP | mmHg |
| Mean Blood Pressure | MBP | mmHg |
| Pulse Pressure | PP | mmHg |
| Heart Rate | HR | bpm |
| Pre-Ejection Period | PEP | ms |
| Left Ventricular Ejection Time | LVET | ms |
| Stroke Volume^A^ | SV | ml |
| Cardiac Output^B^ | CO | l/min |
| Heather Index^C^ | HI | Ohm*s^2^ |
| Systemic Vascular Resistance^D^ | SVR | dyn*s/cm^5^ |
| Total Arterial Compliance^E^ | TAC | ml/mm Hg |
| Thoracic Fluid Content^F^ | TFC | 1/kOhm |
| 1. *Stroke Volume has been calculated using the Sramek and Bernstein formula [18] SV = VEPT*dZmax*LVET/Z0 accounting for weight, height and sex (variable VEPT), Z0 – baseline impedance, dZmax – derivate of Z0 and LVET;* 2. *Cardiac Output has been calculated as CO =SV*HR;* 3. *Heather Index has been calculated as HI = dZmax*TRC (the time interval between the R-peak of the ECG and C-point of ICG wave), characterizing the maximum contraction force of the left ventricle and corresponding to cardiac inotropism;* 4. *Systemic Vascular Resistance has been calculated as SVR = 80*[mean arterial pressure - central venous pressure]/CO, where central venous pressure is assumed 6 mm Hg;* 5. *Total Arterial Compliance has been calculated as TAC = SV/pulse pressure;* 6. *Thoracic Fluid Content has been calculated as TFC = 1000/Z0.* | | |

*
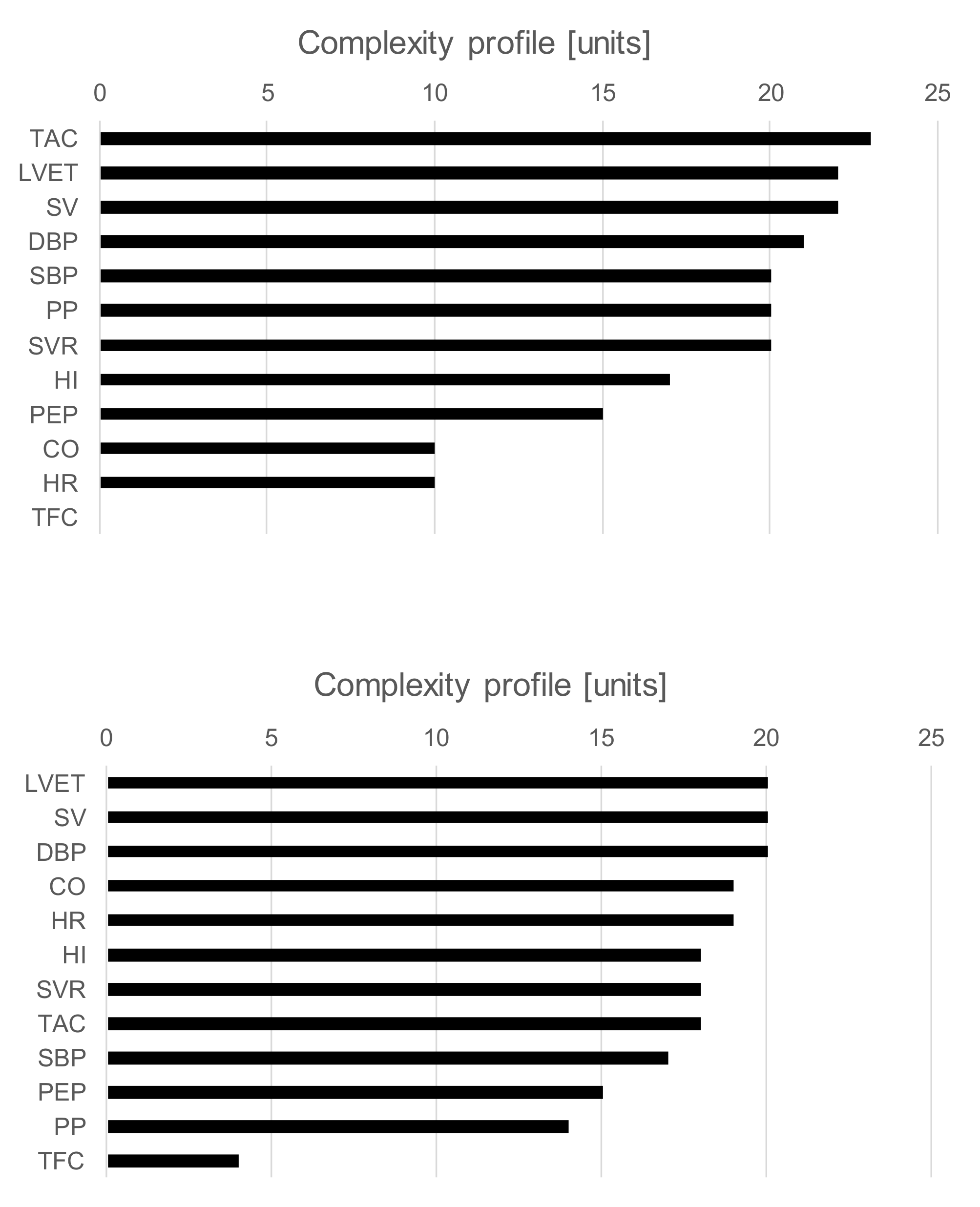
*

Figure S1: Chart a - Complexity profiles at the moment of syncope event for first exemplary subject (S1); chart b - Complexity profiles at the moment of syncope event for second exemplary subject (S2)

Comment: Examples of complexity profiles (CP) are presented in Figure S1. A CP is a breakdown of total system complexity into its components in terms of the percentage of contribution of each component. The contribution of haemodynamic parameters to total complexity is not evenly distributed. The bar chart provides a ranking of haemodynamic parameters in terms of contribution to the vasovagal reaction. For both presented cases, LVET and SV were the top complexity drivers. In contrast, TFC had the lowest contribution. Although both subjects were classified as the mixed type of vasovagal syncope, there were some clear differences between them. For S1, the TAC and blood pressure indices (SBP, DBP, PP) made greater contributions to the vasovagal reaction than HR and CO did, while for S2, the opposite was the case.
